# How many human pathogens are there in Laos? An estimate of national human pathogen diversity and analysis of historical trends

**DOI:** 10.1136/bmjgh-2020-002972

**Published:** 2020-10-22

**Authors:** Madeleine Claire Clarkson, Ricardo Aguas, Kathryn Sweet, Tamalee Roberts, Michel Strobel, Paul N Newton

**Affiliations:** 1 Department of Infectious Disease Epidemiology, London School of Hygiene & Tropical Medicine, London, UK; 2 Lao-Oxford-Mahosot Hospital-Wellcome Trust Research Unit, Microbiology Laboratory, Mahosot Hospital, Vientiane, Lao People's Democratic Republic; 3 Centre for Tropical Medicine and Global Health, Nuffield Department of Medicine, University of Oxford, Oxford, UK; 4 Mahidol Oxford Tropical Medicine Research Unit, Faculty of Tropical Medicine, Mahidol University, Bangkok, Thailand; 5 Independant researcher, Vientiane, Lao People's Democratic Republic; 6 Independant researcher, Toulouse, France

**Keywords:** public health, health systems evaluation, mathematical modelling, epidemiology

## Abstract

**Objective:**

The emergence of infectious diseases pose major global health threats. Estimates of total in-country human pathogen diversity, and insights as to how and when species were described through history, could be used to estimate the probability of new pathogen discoveries. Data from the Lao People’s Democratic Republic (Laos) were used in this proof-of-concept study to estimate national human pathogen diversity and to examine historical discovery rate drivers.

**Methods:**

A systematic survey of the French and English scientific and grey literature of pathogen description in Laos between 1874 and 2017 was conducted. The first descriptions of each known human pathogen in Laos were coded according to the diagnostic evidence available. Cumulative frequency of discovery across time informed the rate of discovery. Four distinct periods of health systems development in Laos were identified prospectively and juxtaposed to the unmodelled rate of discovery. A model with a time-varying rate of discovery was fitted to these data using a Markov-Chain- Monte-Carlo technique.

**Results:**

From 6456 pathogen descriptions, 245 discoveries of known human pathogens in Laos, including repeat discoveries using different grades of evidence, were identified. The models estimate that the Laos human pathogen species diversity in 2017 is between 169 and 206. During the last decade, there has been a 33-fold increase in the discovery rate coinciding with the strengthening of medical research and microbiology.

**Conclusion:**

Discovery curves can be used to model and estimate country-level human pathogen diversity present in a territory. Combining this with historical assessment improves the understanding of the factors affecting local pathogen discovery.

**PROSPERO registration number:**

A protocol of this work was registered on PROSPERO (ID:CRD42016046728).

**Key questions**

**What is already known?**

- Discovery curves have been used to estimate the diversity of diverse taxa such as trees, ants, birds and ferns. They have also been used to estimate global virus species diversity.
- Estimates of country-level pathogen diversity are needed to inform policy on potential of emergence, guide appropriate investment of resources in the skills, facilities and technologies necessary for aetiological discovery, particularly in resource limited setting like Laos.
- A historical analysis of the local context is needed to build on past experience of pathogen discovery to identify factors that facilitate discovery and encourage appropriate investment.

**What are the new findings?**

- From 6455 pathogen description records, 244 first descriptions of known human pathogens in Laos, including repeat discoveries using different grades of evidence, were identified.
- Four distinct periods of health systems in Laos were prospectively identified and juxtaposed to the unmodelled rate of discovery.
- The models estimate that the human pathogen species diversity in 2017 in Laos was between 169 and 206.
- A 33-fold increase in the discovery rate was observed during the last decade, reflecting greatly increased investment in medical research.

**Key questions**

**What do the new findings imply?**

- Our finding show that it is possible, with sparse and historically varied infectious disease surveillance, to model pathogen discovery and estimate pathogen species diversity The time-varying rate discovery curve model is a useful proof-of-concept to estimate how many pathogens may be present in territories with sparse infectious disease surveillance and can be tested prospectively. This type of analysis could be used to guide appropriate investment in resources necessary for aetiological discovery, especially in scarce-resource settings
- Country-level historically linked discovery curve investigations elsewhere could improve our understanding of pathogen diversity between countries and estimate the probabilities of new discoveries and our understanding of the factors influencing such discoveries in different health systems.

## Introduction

Human pathogens are defined as species-level organisms associated with disease or clinical infection in humans.^1^ In 2007, a total of 1399 human pathogen species were described globally.^1^ As far as we are aware there are no maps of variation in national human pathogen species richness, nor predictions of how many pathogens may be present but have not yet been described, globally or in nation states.

The emergence and re-emergence of human pathogens is influenced by numerous demographic, historical, sociological, population and environmental factors.^2^ An increase in spill-over events has prompted research to develop new tools and methodologies for estimating pathogen diversity. There are many methods of varying complexity and associated criticisms for estimating species diversity.^3–9^ The work of the Global Virome Project and others aims to estimate viral diversity to help predict outbreaks, of striking importance in the face of the current pandemic.^10^ However, such work is costly and requires large-scale host sampling. Here, we use existing historical records and discovery curve theory to investigate how many of the globally described human pathogens affect the people of the Lao People’s Democratic Republic (Laos).

Discovery curves have been used to estimate the global diversity of diverse taxa such as trees, ants, birds, ferns and viruses.^3–5^ The method extrapolates from the fraction of species known to exist in a designated area using the rate of discovery of new species over time and propagating that forward into the future until a maximum is reached.^3 11 12^ As far as we are aware, the use of discovery curves to estimate total country-level human pathogen diversity has not yet been attempted.

Laos is a small mountainous land-linked country (~237 955 km^2^), bordered by Thailand and Myanmar to the west, Vietnam to the east, China to the north and Cambodia to the south. Laos’ population is approaching 7 million; the rural population comprises 49 recognised ethnic groups reflecting the social and cultural heterogeneity of the region.^13 14^ The widespread consumption of wild vertebrates in Laos as ‘bushmeat’ and limited rural diagnostic infrastructure increases the likelihood of occurrence, but not the discovery, of zoonotic spill-over events.^15^ Poverty, consumption practises and extreme climates underlie the myriad of diseases that exist in this region.^16 17^

This paper contributes to the ongoing literature on methods for estimating species diversity^4 5^ and used time-varying rate discovery curve modelling of the rate of description of human pathogens in Laos, using Markov-Chain-Monte-Carlo (MCMC) methods, to estimate the total number of human pathogens currently in Laos and understand the historical influences on changes in pathogen within-country discovery rate.

## Methods

Data on human pathogen species and their year of discovery were used to inform the model’s discovery rate. Laos history was categorised into four different phases of health systems to examine changing historical contexts affecting discovery rates (below, online supplemental file, table 1).^8^

10.1136/bmjgh-2020-002972.supp1 Supplementary data

### Search strategy, selection criteria and data structure

As no prior synthesis of the description of human pathogens in Laos was available, a systematic review, using the Preferred Reporting Items for Systematic Reviews and Meta-Analyses guidelines, was conducted (online supplemental file, figure 1). Databases were searched using both French and English search terminology. An iterative search of the grey literature was conducted in Vientiane, Laos, in the Lao Department of National Archives, the National Library, the École française d'Extrême-Orient, WHO Laos National Office library, University of Health Sciences and in Bangkok, Thailand at Mahidol University and in Aix-en-Provence, France, the Archives Nationales d’Outre-Mer. Published e-journals, grey literature and databases were searched of PubMed, the London School of Hygiene and Tropical Medicine library, the South East Asian Journal of Tropical Medicine and Public Health and the Korean Journal of Parasitology and Oxford University databases. The search included publications from 1866, the first French exploration into Laos, and papers published prior to 31 December 2017. Information on disease occurrence using scientific names prior to French colonisation was not found.

All documents referencing the discovery and/or incidence of human pathogens and diseases reported or sampled from Laos were considered, whether in humans, vectors or reservoirs. Documents and searches written in Lao script were excluded due to limited medical standardisation of disease terminology within the language.^18^ The following broad English search terms and their variants were included [“health” OR “medicine” OR “disease” OR “epidemiology” OR “pathology”] AND [“Lao” OR “Laos”] AND [“first” OR “discovery” OR “isolate”]. The following broad French search terms and their variants were included [“santé” OR “pathologie” OR “medecine” OR “maladie” OR “épidémiologie”] AND [“Lao” OR “Laos”]. The grey literature (hospital and medical reports, textbooks and correspondence) that dominated the French language search, meant it was necessary to include not only pathogens identified by authors as first descriptions but also those that we suspected were the first descriptions. In addition, to the systematic electronic and manual searches, references were manually searched when reports were located.

‘Early’ (the period pre-1990s) national health reports, accounts from medical personnel, case reports, journals, theses and other published documents were searched. Only paper health reports from pre-1990 were included as reports after then could be assumed to be identified through internet searches. Using Adobe Professional (V.16.12.13, Adobe Systems) documents were converted into readable-text-format and searched according to the search strategy. Mendeley (Mendeley Desktop 1.16.1, Mendeley) was used to manage references. Uncertain entries were re-examined by PN, TR and collaborators (see the acknowledgements).

Every human pathogen referenced in the source documents was given a new line item in the dataset and accompanied by variables for the date of identification and publication, the source and type of document it came from and a measure of certainty in the diagnosis. Pathogens are defined as potential causes of human disease and are listed in Laos irrespective as to whether they were described as causing disease. Three grades of evidence were used to stratify the dataset into measures of certainty in the discovery specificity.

Grade 1 includes culture and/or molecular assays (which includes PCR diagnosis) and/or direct microscope observation, grade 2 is serology-based diagnosis and grade 3 a clinical diagnosis without aetiological laboratory assays. For most pathogens, grade 3 represents the lowest grade of diagnostic confidence available. The perceived prestige of being the first to identify the presence of a pathogen in Laos may lead to confirmation bias and the graded data helps to account for this.^19^ Under the stratified grading system, the first description was for each the grade of evidence that was used for the diagnosis.

### Statistical and historical analysis

Discovery curves are used to estimate the total number of species in a specified area,^3 11 12 20^ such as used for the diversity of human viral species by Woolhouse *et al*.^1 11 12 21 22^ Discovery curves require that organism taxonomy is generally accepted and assumes that the number of organisms available for discovery is finite.^3^ Hence, as discovery saturates the number of known species approaches the number of existing species.

The geographical region is defined by Laos’ borders that have remained relatively unchanged since the 1893 Siam-France treaty, when Siam (modern Thailand) surrendered territories east of the Mekong to France.^23^ Only one data point, from the town Luang Prabang, was described prior to 1893 and is included in the dataset,^24^ as the town was and remained part of Laos in that year and since.

**Figure 1:**
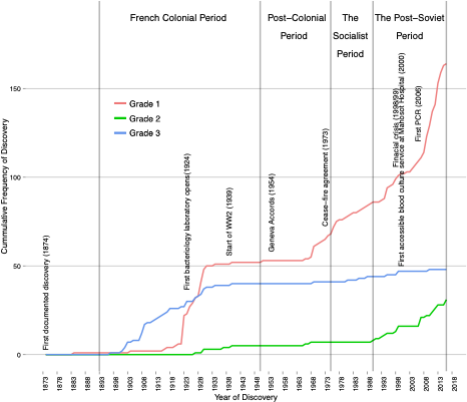
The cumulative frequency of human pathogen discovery in Laos for the period 1874–2017, stratified by the grade of evidence used to identify the pathogens, in which grade 1 represents either culture, direct observation, molecular (which includes PCR diagnosis) or direct microscope observation, grade 2 is serological diagnosis and grade 3 is clinical diagnosis.

Discovery curves comprised cumulative frequency plots of the identified organisms against their year of description.^11 22^ Curves were created by cumulatively plotting organisms against their year of discovery and using interpolation to smooth the results. Three diagnostic technique grade stratified curves formed the backdrop for historical discussion (figure 1). These data were then used to develop three discovery curves of increasing diagnostic certainty.

The first discovery curve (level 3) used the minimum year value (earliest date) from the set containing all grade data. The second curve (level 2) the minimum year value from the set containing only grade 1 and 2 data. The final discovery curve (level 1) used only data from grade 1. Thus the three discovery curves, by incrementally excluding lower quality graded evidence, reflected an increase of certainty in our estimate of pathogen diversity (see online supplemental file, figure 2).

Previous time-based discovery-curve analyses assumed a constant-rate of pathogen discovery. The historical discussion supported a time-variant approach by suggesting four distinct periods of public health experienced in Laos; the French era of colonisation (1893–1953), the postcolonial (1953–1975), the socialist (1975–1990) and the post-Soviet periods (1990 to the present).^13^ The impact of historical events and changing pathogen diagnostic capabilities are functionally unaccounted for if a constant rate of discovery is assumed. We developed a model that used a historical discussion to inform the changing rate of discovery for the years 1873–2017. The model defines the expected number of discovered pathogens in year $t,\lambda_{t}$ so that:

$$\lambda_{t}\left(N,p\right)=Np{\left(1-\overset{-}{p}\right)}^{t-1}$$

The total number of species available for discovery is given by the parameter N. Therefore, when interpreting the model estimate of N to answer the question, ‘how many species of pathogens are there in Laos?’ the sum of pathogens found prior to t=1 c, is added to the estimate. The question posed is answered by N+c.

The probability of discovery at time t is given by parameter p:

$$p(i)=a\left[\frac{1}{1+e^{\left(-0.4\times (i-k)\right)}}\right]+af\left[\frac{1}{1+e^{\left(-0.4\times (i-k_{2})\right)}}\right]+0.002$$

Where the parameters a,k and $k_{2}$ allow for a sigmoidal increase in the probability of discovery and f represents the fold increase of the second inflection point in the rate of discovery. Thus, in this model, the discovery rate changes from its baseline value to a new value at time k and k2.

The parameters were fitted to the data using MCMC Bayesian inference simulation with a standard Metropolis-Hastings parameter sampling algorithm adjustment and a 5000-iteration burn-in period. The MCMC simulations were run for each level of evidence using 1 million runs. All parameters received uniform priors except N and f for which a normal prior distribution was considered reasonable. Mean parameter estimates (after burn-in) were used to compare it to the cumulative distribution of the observed data.

Confidence Intervals around the mean estimates were calculated using the 5th and 95th percentiles of the posterior distributions for each parameter. A sensitivity analysis was conducted using a 1000 bootstrap samples of the posterior parameter distributions (after burn-in) to reflect the estimated uncertainty around the mean estimated model.

### Patient and public involvement

Patients and/or the public were not involved in the design, or conduct, or reporting, or dissemination plans of this research.

## Results

The final pre-exclusion dataset consisted of 6456 entries and the final dataset comprised 5127 entries (figure 1). Each entry included year of discovery, organism name, assigned grade of evidence and reference to the source document (online supplemental file, table 1). After line items had been coded according to date of appearance for each grade, these data were further reduced to 244 species-level discoveries, including repeat discoveries at different grades of evidence (for a complete list of first descriptions see online supplemental file, table 1). The final dataset spanned the period 1874–2017. No human pathogens have yet been described for the first time as new species to science in Laos.

The year of description and grade of evidence for each of the 244 identified species was used to develop the grade stratified figure 1, as well as the discovery curves in online supplemental file, figure 2; The shapes generated for grade 1 in these figures, even with deceleration in the discovery rate from 2013, do not suggest a clear trend towards an asymptote, indicating discovery has not yet saturated.

**Figure 2:**
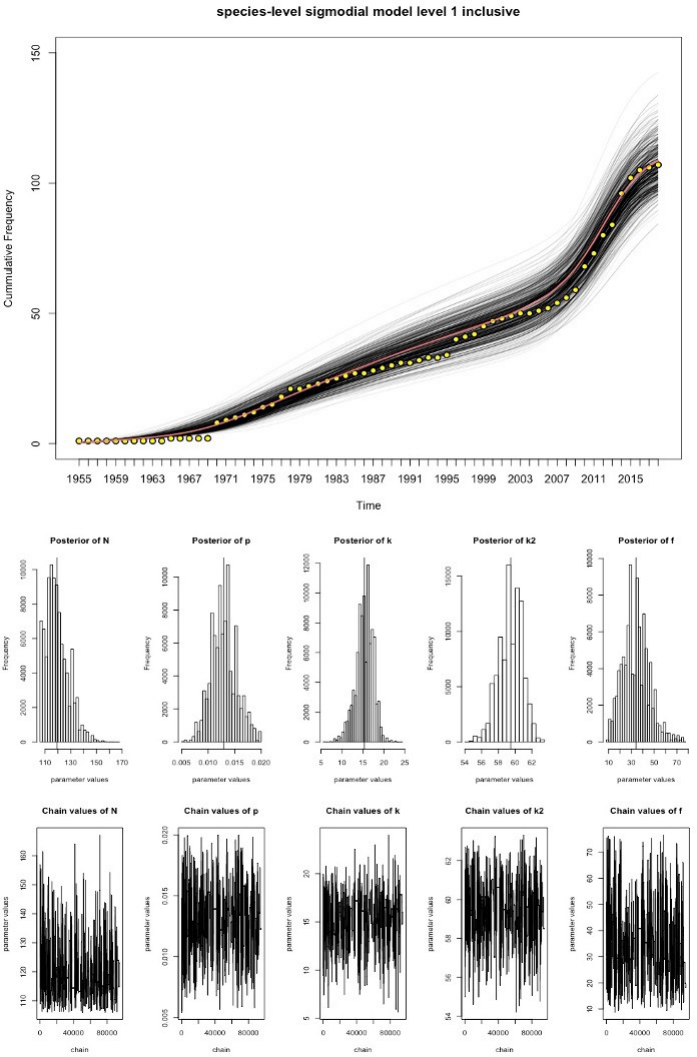
Discovery curve model for level 1 data and MCMC output the yellow points represent the observed data, the red line is the best fitting model and the black lines represent the uncertainty in the model estimates. Underneath the model fit curve are a series of histograms which represent the posterior distributions of parameter estimates. The chain output can be seen at the bottom of the figure and represent the range of parameter values tested and the number of model iterations. MCMC, Markov-Chain-Monte-Carlo.

**Figure 3:**
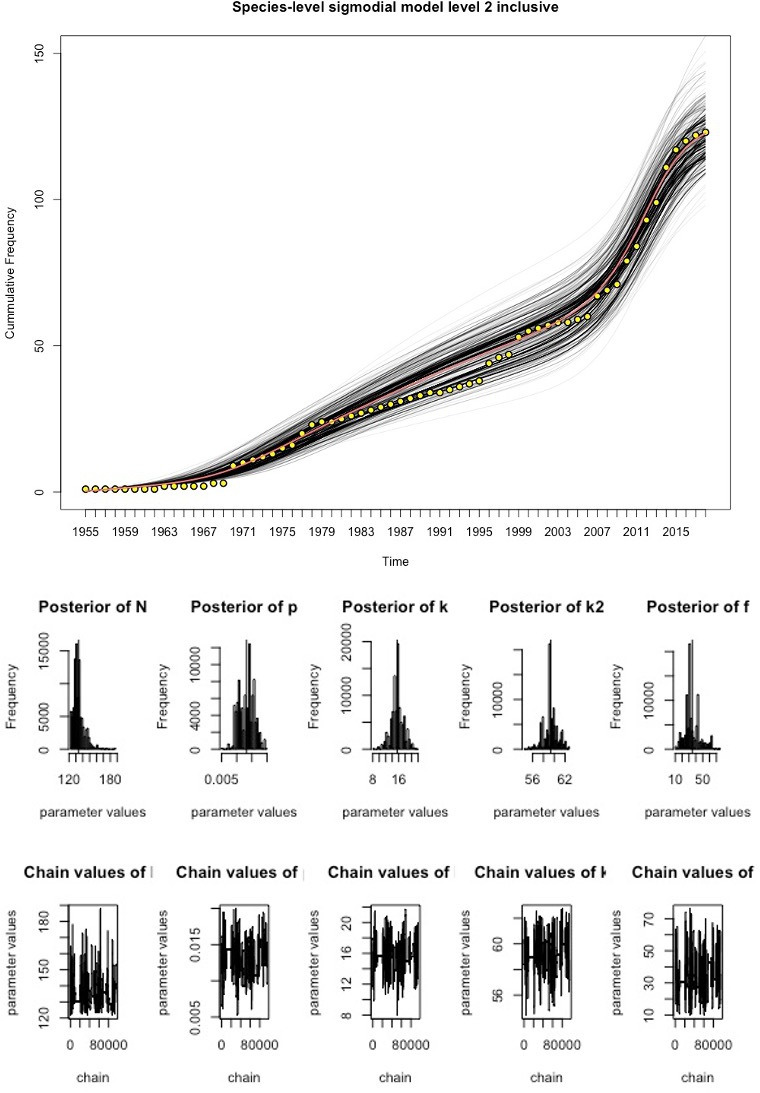
Discovery curve model for level 2 data and MCMC output. The yellow points represent the observed data, the red line is the best fitting model and the black-lines represent the uncertainty in the model estimates. Underneath the model are a series of histograms which represent the posterior distributions of parameter estimates. The chain output can be seen at the bottom of the figure and represent the range of parameter values tested and the number of iterations. MCMC, Markov-Chain-Monte-Carlo.

**Figure 4:**
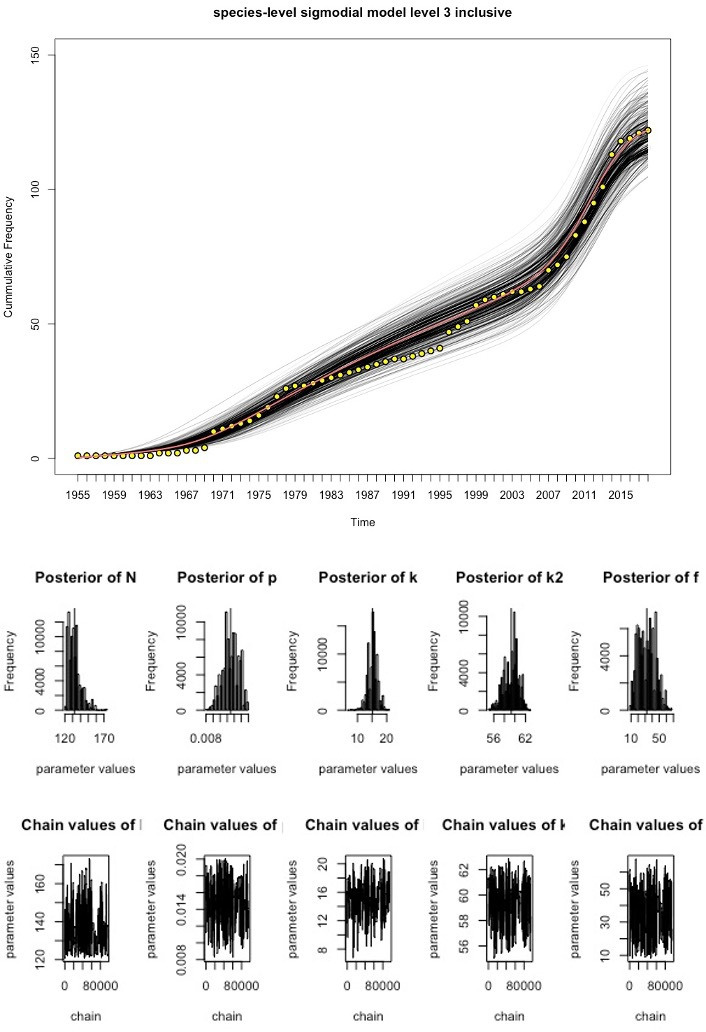
Discovery curve model for level 3 data and MCMC output. The yellow points represent the observed data, the red line is the best fitting model and the black lines represent the uncertainty in the model estimates. Underneath the model are a series of histograms which represent the posterior distributions of parameter estimates. The chain output can be seen at the bottom of the figure and represent the range of parameter values tested and the number of iterations. MCMC, Markov-Chain-Monte-Carlo.

We fitted the time-varying discovery rate model to the cumulative curves in figure 1 to estimate the total number of existing pathogen species in Laos for each grade of evidence, using MCMC techniques (figures 2–4, table 1). As the observed sustained flattening of the curves in the postcolonial era (figure 1) could not be modelled, the models were restricted to years after 1953. The discoveries before 1954 were captured by a constant (ie t=1 is the year 1954). All the models and parameters closely fit the data with approximately normal distributions (figures 3–4). Table 1 compares estimated parameters across the models. The estimates of 169 and 206 (table 1), N+c, are the first approximations of pathogen diversity in Laos. Where c is the number of discovered pathogens at the 1954 time point, and N is the estimate derived from modelling the data post-1954. The fold increase, f, of the discovery rate following the last inflection are 33.99, 32.30 and 34.83 for the levels 1, 2 and 3 models, respectively.

**Table 1:** Summary of estimates and parameter confidence intervals by model

Parameters	Level 1 discovery curve model (Includes only grade 1 evidence of discovery)	Level discovery curve model (Includes grade 1 or 2 evidence of discovery)	Level 3 discovery curve model (Includes grade 1, 2 or 3 evidence of discovery)
Total no of species known in 2017 (unmodelled)	156	173	195
c	50	51	74
N+c	169	185	206
N	119.36 (107.50–134.98)	134.07 (123.98–148.55)	132.29 (123.12–147.48)
p	$p(a,k,k_{2},f)$	$p(a,k,k_{2},f)$	$p(a,k,k_{2},f)$
a	0.013 (0.009–0.017)	0.013 (0.009–0.017)	0.015 (0.011–0.019)
k	15.35 (11.52–18.47)	15.56 (12.28–18.96)	15.18 (12.50–17.79)
$k_{2}$	59.47 (57.05–61.66)	59.33 (57.32–61.45)	59.32 (56.91–61.45)
f	33.99 (15.86–54.52)	34.83 (16.91–59.88)	32.30 (15.03–53.16)

## Discussion

The changing diagnostic capabilities of Laos are reflected in the granularity of pathogen discoveries over time. The majority of human pathogens recorded from Laos to date are bacteria (online supplemental file, table 2). Bacteriological techniques (grade 1) and limited serology (grade 2) arrived in Laos in the late 19th century and began to replace clinical descriptions (grade 3), while recently the molecular technologies have begun to predominate in the literature (figure 1).^25^

Over the last 150 years Laos’ path of pathogen discovery has been disrupted by political unrest and conflict and undergone an ideological transformation from a loosely ruled monarchy to a socialist state (figure 1). Mapping these histories to the discovery curve provides a step towards understanding the complexities that shape pathogen discovery and modelling the rate of discovery.

The first written French description of a pathogen in Laos was in 1874 (a cholera outbreak), recorded in a 1904 French publication.^24^ From 1893 the gradual increase in grade 3 diagnoses could be ascribed to the expansion of early French healthcare and associated clinical descriptions of the diseases encountered. Under French colonial rule the numbers of hospitals with medically trained staff increased from 0 to 12.^13^ The French setup basic training and medical facilities, including a small bacteriology laboratory (in 1924) at Mahosot Hospital, Vientiane.

An increase in laboratory capabilities is matched by a decline in the frequency of clinical discovery (grade 3). Post-1924 laboratory developments include, enhanced microscopical capacities (a grade 1 recognised level of evidence) and limited serological testing (grade 2), influencing the shapes of grades 1 and 2 curves, respectively (figure 1). During the French colonial occupation of Laos, there was a limited rural health worker presence and therefore while Laos did have some expertise and facilities necessary for aetiological investigation, these resources were limited and inaccessible for much of the country.^13^ French funding and support decreased in the mid-1930s and during World War 2.^13^ The effects of which are reflected in a tapering off of the all-grade discovery rates (figure 1). In 1950, control of the health facilities was transferred to Lao authorities.

During the postcolonial period the all-grade flattening of the discovery curves continued until the late 1960s and early 1970s. Early into this period two competing forms of nationalism emerged in Laos: the Royal Lao Government (RLG) and the prosocialist Pathēt Lao.^13^ The Geneva Accords of 1954 were an attempt to bring stability to the region through the partitioning of the country between these groups.^26^ However, the inherited health facilities, funding and skills were unevenly split between these partitions. The colonial health facilities were transferred to the RLG and complemented by a modest emerging private healthcare sector run by RLG staff. Alongside these local health facilities, US and Philippino aid-agencies set up a network of rural hospitals and medical dispensaries.^13^ However, the Pathēt Lao was responsible for the majority of the rural areas, where they set up a network of rudimentary military health facilities.^13^ Therefore, the flattening of the discovery curves in figure 1 likely reflects a decrease in aetiological investigation during this period of discord.

An increasing foreign presence and Cold-War conflicts amplified the political divisions within Laos and in 1975, the USA, who had supported the RLG, left Laos. The Pathēt Lao gained political control of the country, heralding the socialist period.^13 26^

A Soviet model of socialism was maintained from 1975 until the 1990s. The adopted Soviet-model of primary healthcare was set up to provide free nationwide healthcare.^13^ After the regimen change many of the medical staff, linked to the RLG, left the country resulting in a deficit of already limited skilled medical professionals. An increased coverage and centralisation of authority tempered by a decreased skills capacity could explain the observed more gradual incline in the grade 1 discovery curve (figure 1). During the socialist period, the Lao government received support from other socialist states. As this economic structure began to collapse internationally, so did the support that they provided to nations like Laos.

In the 1990s, Laos incorporated elements of free-market trade into policy, creating a new era of post-Sovietism.^13 26^ Despite these changes, Laos’ contributions to scientific literature during the 1990s remained low.^27^ Investment in diagnostic capabilities increased in 1998/1999,^13 27^ reflected in the steep incline of grade 1 data (figure 1). Since the introduction of PCR assays for patient diagnosis in 2006, at Mahosot Hospital, an increasing number of human pathogens have been detected by PCR assays (figure 1), reflected in a steep incline of grade 1 discovery curves in the 2000s.

This changing politics and health of Laos forms a useful backdrop for the developed models. In the context of Laos narrative the plateauing off of the curves suggests that French colonial era years had a limited impact on the future discovery rate. However, this era was still responsible a large proportion of, mostly clinical, early first descriptions of pathogens.

These early clinical descriptions are still important as in some instances, for example, Epstein-Barr virus in 1966 or chicken pox virus in 1927,^28 29^ they represent the only descriptions of the presence of these pathogens in Laos. To recognise the importance of these early discoveries the models used the grade of evidence to create the data subsets (levels) which were then used to inform the three models. The discovery curve in model 3 includes clinical diagnoses (grade 3) as first descriptions. Clinical diagnosis is usually not as definitive as other technological advancements. Therefore, model 2 excludes clinically described pathogens as first discoveries. Similarly, model 1 excludes serologically or clinically described pathogens.

The estimates from the models suggest that between 11 (level 3), 12 (level 2) and 13 (level 1) new species are currently available for discovery in Laos, reflecting estimated total species diversity of 169, 185 and 206, respectively (table 1). Although 244 descriptions were recorded, these included repeat ‘first’ descriptions at different grades. These represent 195 different species with any grade of positive evidence. As the discovery curves used in the models did not permit repetition, estimates are expected to be lower than the total number of descriptions. A sensitivity analysis shows a range of diversity estimates of between: 157–185 for level 1, 175–200 for level 2 and 197–221 for level 3. Although there are no other estimates with which to compare these, this proof of concept provides a basis for future evaluation.

The differences in the estimated diversity are greatest between the levels 1 and 3 models, due in part to c, the number of pathogens discovered before 1954. The level 3 data includes many clinical descriptions that have not been described at a higher grade of evidence and are subsequently not reflected in the modelled data for higher levels. For example, the clinical specificity of some diseases, such as rabies, together with the past and current aetiological diagnosis limitations of Laos, has inhibited the description of their pathogens at higher published evidence grade. The history of foreign involvement and dependence has meant that Laos has had a limited number of locally trained technically skilled staff and necessary diagnostic capabilities for further aetiological investigation until ~2000.

Although there is a 16 species difference between the level 2 and level 1 models, there is only a difference of 1 between their baseline c value, suggesting that the main differences occur in the actual model. Given the current capacity for health research in Laos, model 2 (inclusive of grade 2 evidence) is likely to represent the expected future trajectory in discovery, depending on serological diagnosis, unless significant nationwide investment in molecular and culture methods are made.

Despite a slight decline in rate of discovery in the second decade of this century, the models are not asymptotic, suggesting that data informing the model is incomplete and/or that the key modelling assumption of a finite number of pathogens available for discovery in Laos is not met. Accounting for emergent and imported pathogens will be crucial for further modelling of these data.

The majority of emergent zoonotic diseases are viral, of which there are comparatively fewer recorded discoveries in Laos (online supplemental file, table 2).^30^ Although bats are major reservoirs of human disease,^31^ there are no current publications on bat-human pathogens in Laos suggesting a possible source of future emergence and discovery.

The gradient change is captured in the model by the parameter, f, and gives an indication of the recent effort associated with pathogen description in Laos. The 34-fold increase in the discovery rate over the last 10 years in model 1 (table 1) suggests that recent efforts and technological advancements have had a significant impact on the discovery of pathogens and it will take more infectious disease research before the number of pathogens available for discovery saturates.

Important limitations of this study include the exclusion of languages other than French and English, and not searching archives in Vietnam and Russia. The use and definition of species represents difficulty for some pathogens, such as for *Salmonella enterica* that includes both typhoid and non-typhoidal Salmonella. This research does not investigate the risk of associated morbidity or mortality predicted with the emergence of new infections. However, there is potential to use these data to develop a model that considers the evolutionary stages, as identified by Wolfe *et al*,^32^ to predict if an emergent infection is likely to persist in the population causing long term mass morbidity/mortality or is self-limiting within human populations.

Despite the recent increase in pathogen discovery rate, Laos has among the lowest health-science outputs of countries in the Association of South East Asian Nations.^27 33^ While the 34-fold increase (using model 1 outputs) can be attributed to the recent investments in human capacity and techniques, these advancements reflect a continued dependence on international collaborations.^34^ With expenditure on health low (2.8% of gross domestic product in 2015), the health sector remains underdeveloped for discovery and surveillance of new pathogens.^35^

Despite the uncertainties in the model, the observed recent great increase in the discovery rate should inform national policy and investment, to understand what human pathogens are present, and how much further investment in pathogen description is needed, to ensure that strategies are in place to protect public health. Accompanying these investigations with local historical analysis builds on past experience of pathogen discovery to identify factors that influence discovery and encourage investment. Comparative country-level historically linked discovery curve investigations could improve our understanding of diversity variability, estimate the probabilities of new descriptions and our understanding of the factors influencing such discoveries in different health systems.
