# Supplementary material for: How many human pathogens are there in Laos? An estimate of national human pathogen diversity and analysis of historical trends
Source: BMJ Glob Health. 2020 Oct 22;5(10):e002972. doi: 10.1136/bmjgh-2020-002972 (PMC7584012; doi:10.1136/bmjgh-2020-002972)
Supplement: Supplementary data [file bmjgh-2020-002972supp001.pdf]

SUPPLEMENTARY FILE

Madeleine C Clarkson, Ricardo Aguas, Kathryn Sweet, Tamalee Roberts, Michel Strobel, Paul N Newton. How many human pathogens are there in Laos? An estimate of national human pathogen diversity and analysis of historical trends

A. Additional Methods

1. Exclusion Criteria

Entries were excluded if: the disease or organisms was not specific to the species level; if the methodology used for the diagnosis was unclear and therefore could not be assigned an appropriate evidence grade; if the outcome of tests or examinations were unclear or returned a negative result; if the discovered organism was known not to be an agent of human infection or disease; if a symptom was described but could not be traced to a single causative organism (specifically referencing clinical diagnoses); if the organism could not be traced to current taxonomic practise or the causative agent could not be derived from the translation (unclear handwriting and or missing text). Reports of infectious diseases among emigrants, travellers and refugees were excluded as there is uncertainty whether the pathogen was contracted in Laos.

2. Notes on Lao Pathogen Line List Issues

Data line items were individually considered for eligibility on the basis of: likelihood of presence of pathogen, specificity of disease, pathogenic nature of organism (either identified as non-pathogenic or non-pathogenic to humans), taxonomic level available, a likely misspecification or contaminant, unknown sample specimen or a specimen collected outside of Lao PDR. The complied dataset was reviewed multiple times by MCC and PNN. Suspicious line items were followed up by a re-examining of the reference article and if need be further research or specialist consultation.

a. Unlikely Organisms

A line item that was excluded on the basis of likelihood was the *Leishmania* genus. The report came from the annual national RLG hospital report of 1966 (1). In the report numbers of "consult" referring to patients was listed as 644 and was attended by "consultations" amounting to 1,351. However, no other information is provided, and patients were not

Clarkson MC, *et al. BMJ Global Health* 2020; 5:e002972. doi: 10.1136/bmjgh-2020-002972

admitted nor received any days of treatment. The laboratory report at the end of the document gave no confirmatory evidence.

Historically there have been investigations into the presence of *Leishmania* in Laos. A report from 1937 documented a negative report resulting from an investigation of spleen smears of dogs from Vientiane for the presence of *Leishmania* (2). In even earlier reports, "Kala azar" was mentioned in Rapport Medical Mensuel from 1923 and 1924 which indicate it was being at least looked for in what we presume is now the Microbiology Laboratory at Mahosot Hospital, but without any details of what laboratory tests were performed or clinical details it is impossible to confirm this diagnosis (3,4). A 2012 journal article (5) and searches of Pubmed and Google Scholar revealed no further evidence of *Leishmania* in Laos. With this uncertainty we decided not to record this disease as present in Laos, However, *Leishmania* is an emergent threat in this region with a growing number of case reports from its neighbour, Thailand, suggesting that future discoveries are likely (6).

The other line item excluded on this basis, was the Ross River virus. This appeared as a short paragraph in a French publication (7). There was insufficient information in the publication to source the original report. Subsequent searching of Pubmed and Google Scholar suggested that Ross River virus is not known to be in Lao or in any adjacent countries.

b. Unclear diagnosis

An unclear diagnosis was one where either the report was misleading or did not provide detail on the level of the diagnosis or for which no clear inference could be made, as per example, " 1 cas de spirochetose pulmonaire gueri par une injection intramusculaire" appeared in a 1920 report but the specific causative agent is unclear (8).

c. Misidentification

The first reports of schistosomiasis in Lao were as early as 1935 (9). The species that was recorded was stated to be *Schistosoma mansoni*. Later reports identify the species as *Schistosoma japonicum*. It was only in 1973, in a paper by Snormani et al., that is was first described as a separate species of a "japonicum-like" appearance (10). The "japonicum-like" species was given the name *Schistosoma mekongi* in 1978. The identification of the species is recognized in these data as 1973, as "japonicum-like" implies a distinct and separate organism (11). *Schistosoma mekongi* is clinically similar to *Schistosoma mansoni* and

Schistosoma japonicum, suggesting that the records of these two *Schistosoma* species were a consequence of misidentification (12).

Isospora hominis and *Sarcocystis hominis* were recorded as separate organisms in a paper by Giboda et al. (13). *Isospora hominis* was subsequently identified as a separate stage of the organism *Sarcocystis hominis* (14). This implies that Giboda et al. double reported the same organism as separate pathogens, resulting from the previous misclassification.

d. Non-pathogenic in humans

For the purpose of this paper the Oxford English Dictionary definition of a pathogen was applied, which states (15): “A microorganism that can cause disease”

A combination of PubMED, Google Scholar and textbook searches were used to determine the pathogenic nature of organisms (16). Within the items not meeting the eligibility criterion of pathogenic organisms were: *Phthiriasis pubis* and *Pediculus humanus*. These organisms were excluded on the basis of not meeting the definition of being agents of disease. Pubic and head lice are associated with discomfort but we decided not to regarded as causing disease.

However, the anthropods *Sarcoptes scabiei*, *Chrysomya bezziana* and *Demodex* spp. all meet the disease eligibility criterion and are included in the analysis, because they are associated with subsequent pathology post-inoculation.

e. Unspecified organisms associated with a disease, syndrome or pathogen description

A number of the hospital reports and early documentation listed a disease, syndrome or pathogen description. For many diseases, syndromes and pathogen descriptions are sufficient to identify singular causative agent, such as is the case with measles that is caused solely by the measles virus. Two descriptions of herpes simplex virus (HSV) 1 and 2 were categorised by their clinical manifestation (HSV1 is an oral and HSV2 is a sexually associated infection (17)). However, a number of clinical and phenotypic descriptions are common to multiple organisms. The report of “hookworm” in the data, which can refer to multiple organisms, was a frequent example of this. Line items of this nature were excluded, as it was not specific enough to allow for inference as to the causative organism.

d. Probable contaminants

Probable contaminants were identified by the isolate sample and the clinical features by PNN.

e. Samples collected outside Laos

These reports were excluded during the selection of appropriate literature. as, inclusion of samples collected outside of Lao invites uncertainty into the likelihood of the pathogen being present in Lao. There is no way to be sure that the organisms identified were not acquired elsewhere. Temporary resettlement is identified as a contributing factor to communicable disease, making refugees more susceptible to diseases acquired as a result of the displacement (18).

f. Unknown samples

A number of reports cited organisms without the sample from which they were isolated. Often the isolates were recorded as being sampled from "Divers" which translates from French to English as “various”. Without this information it is not possible to assign a grade for the strength of evidence. Since the strength of evidence was the basis of the analysis these organisms were excluded from the analysis.

References

1. Royaume du Laos ministére de Santé publique. Rapport Statistique de L’assistance Médicale. Vientiane; 1966.
2. Lefebvre. Recherches sur le Paludisme au Laos. Rev Med fracaise d’extreme orient. 1937;336–51.
3. Assitance medicale Indochine. Rapport Medical Mensuel de Juillet 1923. 1923.
4. Assitance medicale du Laos Indochine. Rapport annuel 1924. 1924.
5. Viroj W. Leishmaniasis in Southeast Asia: The story of the emergence of an imported infection in a non-endemic area of the world. J Univ Malaya Med Cent. 2012;15(1):1–4.
6. Chusri S, Hortiwakul T, Silpapojakul K, Siriyasatien P. Case report: Consecutive cutaneous and visceral leishmaniasis manifestations involving a novel Leishmania species in two HIV patients in Thailand. Am J Trop Med Hyg. 2012;87(1):76–80.
7. Cattin O. LAOS : UN PAYS ENCLAVE. Med Trop. 2000;60:121–4.
8. Assitance medicale Indochine. Rapport Medical Mensuel d’ensemble 1920. 1920.
9. Annual Report of Assistance Medicale du Laos. Paris; 1935.
10. Sornmani S, Schneider C, Kitikoon V. Life Cycles of Schistosoma Japonicum-Like Trematode From Khong Isand Southern Laos. South East Asian J Trop Medicne Public. 1973;4(2):279-?
11. Voge M, Bruckner D, Bruce JI, Voge, M., Bruckner, D., & Bruce J. Schistosoma mekongi sp. n. from Man and Animals, Compared with Four Geographic Strains of Schistosoma japonicum. J Parasitol [Internet]. 1978;64(4):577–84. Available from: http://www.jstor.org/stable/3279936

12. Ohmae H, Sinuon M, Kirinoki M, Matsumoto J, Chigusa Y, Socheat D, et al. Schistosomiasis mekongi: From discovery to control. Parasitol Int. 2004;53(2):135–42.

13. GIBODA M, Viengsay M, Bouaphan S, Ditrich 0. EPIDEMIOLOGIE DES PARASITOSES INTESTINALES AU LAOS. Bull Société Pathol Exot. 1991;84:184–93.

14. Palmer SR, Soulsby, Lord, Torgerson P, Brown DW. Oxford Textbook of Zoonoses: Biology, clinical practice, and public health control. second. Oxford: Oxford University Press; 2011.

15. OED Online. “pathogen, n.” [Internet]. Oxford University Press. 2016 [cited 2016 Aug 28]. Available from: http://www.oed.com/view/Entry/138792?redirectedFrom=pathogen%5D

16. Mandell GL, Bennett JE, Dolin R. PRINCIPLES AND PRACTICE OF INFECTIOUS DISEASES. 7th ed. Philadelphia: Elsevier Ltd; 2010.

17. Heymann DL. Control of Communicable Diseases Manual. 20th ed. Ross E, editor. Washington DC: Apha Press; 2015.

18. Connolly M, Gayer M, Ryan M, Salama P, Spiegel P, Heymann D. Communicable diseases in complex emergencies: impact and chllenges. Lancet. 2004;364(9449):1975–83.

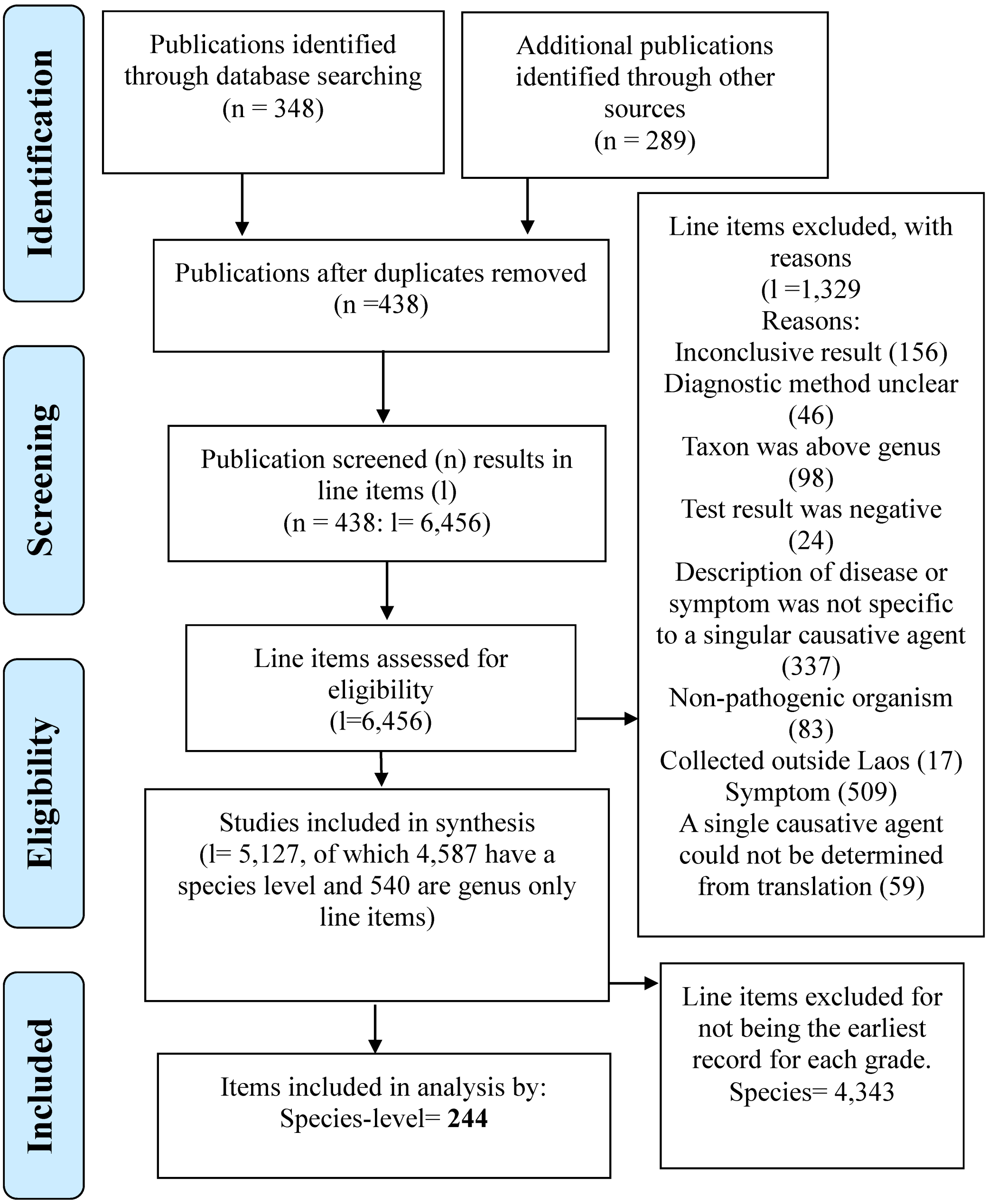

Figure 1: PRISMA diagram

Table 1 List of pathogens, the year of discovery in Laos and the grade of evidence.

Year of discovery	Species name	Grade of strength of evidence used to make diagnosis
2011	*Acinetobacter baumannii*	1
2011	*Actinomadura madurae*	1
1925	*Actinomyces israelii*	3
1994	*Aeromonas sobria*	1
1929	*Alcaligenes faecalis*	1
2013	Alpha coronavirus (HCoV-229E)	1
2013	Alpha coronavirus (HCoV-NL63)	1
1989	*Ancylostoma ceylanicum*	1
1923	*Ancylostoma duodenale*	1
1971	*Angiostrongylus cantonensis*	1
1909	*Ascaris lumbricoides*	3
1923	*Ascaris lumbricoides*	1
1900	*Bacillus anthracis*	3
1929	*Bacillus anthracis*	1
1929	*Balantidium coli*	1
2009	*Bartonella clarridgeiae*	1
2008	*Bartonella henselae*	2
2013	Beta coronavirus (HCoV--HKU1)	1
2013	Beta coronavirus (HCoV-OC43)	1
2012	*Brucella abortus*	2
1999	*Burkholderia pseudomallei*	1
1998	*Campylobacter coli*	1
2007	*Campylobacter fetus*	1
1998	*Campylobacter jejuni*	1
1929	*Candida albicans*	1
1997	*Candida albicans*	3
1999	*Capillaria philippinensis*	1
1970	*Centrocestus formosanus*	1
1977	Chikungunya virus	1
1994	Chikungunya virus	2
1922	*Chlamydia trachomatis*	3
2006	*Chlamydia trachomatis*	1
2008	*Chromobacterium violaceum*	1
1936	*Chryseobacterium indologenes*	1
1928	*Chrysomya bezziana*	3
1941	*Chrysomya bezziana*	1
1923	*Clonorchis sinensis*	1

1937	*Clonorchis sinensis*	3
2017	*Clostridium difficile*	1
1918	*Clostridium tetani*	3
2015	*Clostridium tetani*	2
1906	*Corynebacterium diphtheriae*	3
1931	*Corynebacterium diphtheriae*	1
2015	*Corynebacterium diphtheriae*	2
2010	*Coxiella burnetti*	2
2005	*Cyclospora cayetanensis*	1
2013	*Cytoisospora belli*	1
1911	Dengue virus	3
1992	Dengue virus	2
2010	Dengue virus	1
1929	*Dicrocoelium dendriticum*	1
2009	*Diphyllobothrium latum*	1
1974	*Diphyllobothrium mansoni*	1
1977	*Dipylidium caninum*	1
2005	*Echinochasmus japonicus*	1
1928	*Echinococcus granulosus*	3
1975	*Echinococcus granulosus*	1
2012	*Echinostoma macrorchis*	1
2012	*Echinostoma malayanum*	1
2012	*Echinostoma revolutum*	1
1914	*Entamoeba histolytica*	3
1923	*Entamoeba histolytica*	1
2011	*Enterobacter aerogenes*	1
1995	*Enterobacter cloacae*	1
1909	*Enterobius vermicularis*	3
1923	*Enterobius vermicularis*	1
2011	*Enterococcus faecalis*	1
2009	Enterovirus A	1
1966	Epstein-Barr virus	3
1914	*Escherichia coli*	3
1927	*Escherichia coli*	1
1977	*Exophiala jeanselmei*	3
1989	*Fasciola gigantica*	1
1930	*Fasciola hepatica*	1
1930	*Fasciolopsis buski*	1
1923	*Fusobacterium necrophorum*	1
1923	*Giardia intestinalis*	1
1974	*Gnathostoma spinigerum*	1
1926	*Haemophilus aegyptus*	1
1907	*Haemophilus ducreyi*	3

1923	*Haemophilus ducreyi*	1
1927	*Haemophilus influenzae*	1
1989	*Haplorchis pumilio*	1
1989	*Haplorchis taichui*	1
1989	*Haplorchis yokogawai*	1
2000	*Helicobacter pylori*	1
1998	Hepatitis A virus	2
1965	Hepatitis B virus	2
2008	Hepatitis B virus	1
1998	Hepatitis C virus	2
2008	Hepatitis C virus	1
1998	Hepatitis E virus	2
2010	Hepatitis E virus	1
1997	Herpes simplex virus 1	3
2008	Herpes simplex virus 2	3
1927	Human alphaherpesvirus 3	3
2013	Human bocavirus	1
1989	Human immunodeficiency virus (unspecified)	2
2010	Human Metapneumovirus	1
2012	human papillomavirus	1
1925	Human papillomavirus	3
2013	Human Respiratory Syncytial virus	1
2009	*Hymenolepis diminuta*	1
1991	*Hymenolepis nana*	1
1900	Influenza A virus	3
2007	Influenza A virus	1
2010	Influenza A virus	2
2007	Influenza B virus	1
2013	Influenza C virus	1
1977	Japanese encephalitis virus	1
1989	Japanese encephalitis virus	2
1992	Japanese encephalitis virus	3
1995	*Klebsiella oxytoca*	1
1930	*Klebsiella pneumoniae*	1
1997	*Leptospira interrogans*	2
2015	*Leptospira interrogans*	1
2011	*Listeria monocytogenes*	1
1929	*Macracanthorhynchus hirudinaceus*	1
1923	*Malassezia furfur*	1
1906	Measles virus	3
2011	Measles virus	2
1927	*Moraxella lacunata*	1
1959	*Morganella morgani*	1

1906	Mumps virus	3
2014	Mumps virus	1
2012	*Mycobacterium bovis*	2
1903	*Mycobacterium leprae*	3
1917	*Mycobacterium leprae*	1
1900	*Mycobacterium tuberculosis*	3
1920	*Mycobacterium tuberculosis*	1
1936	*Mycobacterium tuberculosis*	2
1924	*Necator americanus*	1
1900	*Neisseria gonorrhoeae*	3
1923	*Neisseria gonorrhoeae*	1
1926	*Neisseria meningitidis*	1
1926	*Neisseria meningitidis*	3
2006	*Neorickettsia sennetsu*	1
2015	*Nocardia aobensis*	1
1977	*Nocardia brasiliensi*	1
1930	*Opisthorchis felineus*	1
1968	*Opisthorchis viverrini*	1
1930	*Orientia tsutsugamushi*	3
1938	*Orientia tsutsugamushi*	2
2015	*Orientia tsutsugamushi*	1
2013	*Paragonimus bangkokensis*	1
1997	*Paragonimus harinasutai*	1
1969	*Paragonimus heterotremus*	1
2013	*Paragonimus paishuihoensis*	1
1969	*Paragonimus westermani*	1
2013	Parainfluenza virus 1	1
2010	Parainfluenza virus 3	1
2013	Parainfluenza virus 4	1
2005	*Penicillium marneffei*	1
2009	*Phaneropsolus bonnei*	1
1977	*Phialophora pedrosoi*	1
1925	*Plasmodium falciparum*	1
1925	*Plasmodium malariae*	1
1997	*Plasmodium ovale*	1
1923	*Plasmodium vivax*	1
1928	Poliovirus unspecified	3
1966	Poliovirus unspecified	1
1966	Poliovirus unspecified	2
2009	*Prosthodendrium molenkampi*	1
1995	*Proteus mirabilis*	1
1995	*Proteus vulgaris*	1
1931	*Pseudomonas aeruginosa*	1

1906	Rabies virus	3
1929	Rabies virus	1
1929	Rabies virus	2
2010	Respiratory Syncytial virus (RSVA)	1
2006	*Rickettsia conorii*	2
2006	*Rickettsia felis*	2
2009	*Rickettsia felis*	1
2006	*Rickettsia helvetica*	2
2016	*Rickettsia japonica*	2
1907	*Rickettsia Prowazekii*	3
2006	*Rickettsia tamurae*	2
2006	*Rickettsia typhi*	2
2015	*Rickettsia typhi*	1
1922	Roseolovirus	3
1907	Rubella virus	3
2011	Rubella virus	2
1909	*Salmonella enterica*	3
1925	*Salmonella enterica*	1
1929	*Salmonella enterica*	2
1989	*Sarcocystis hominis*	1
1904	*Sarcoptes scabiei*	1
1973	*Schistosoma mekongi*	1
1989	*Schistosoma spindale*	1
2011	Seoul virus	1
1977	*Shigella boydii*	1
1994	*Shigella dysenteriae*	1
1969	*Shigella flexneri*	1
1969	*Shigella sonnei*	1
1977	Sindbis Virus	1
2014	*Spirometra erinaceieuropaei*	1
2005	*Sporothrix schenckii*	1
1969	*Staphylococcus aureus*	1
1977	*Staphylococcus aureus*	3
1995	*Staphylococcus epidermidis*	1
1977	*Staphylococcus epidermidis*	3
1995	*Staphylococcus saprophyticus*	1
1989	*Stellantchasmus falcatus*	1
2011	*Streptococcus agalactiae*	1
1907	*Streptococcus group A*	3
2009	*Streptococcus mutans*	1
1923	*Streptococcus pneumoniae*	1
1929	*Streptococcus pneumoniae*	3
1915	*Streptococcus pyogenes*	3

1930	*Streptococcus pyogenes*	1
2014	*Streptococcus suis*	1
1923	*Strongyloides stercoralis*	1
2009	*Taenia asiatica*	1
2010	*Taenia hydatigena*	1
1923	*Taenia saginata*	1
1930	*Taenia solium*	1
1930	*Toxocara canis*	1
1989	*Toxocara cati*	1
2014	*Toxocara vitulorum*	1
1992	*Toxoplasma gondii*	2
1901	*Treponema pallidum*	1
1926	*Treponema pallidum*	2
1900	*Treponema pallidum*	3
1923	*Treponema vincentii*	1
1973	*Trichinella spiralis*	1
1969	*Trichomonas vaginalis*	1
1908	*Trichophyton concentricum*	3
1923	*Trichophyton concentricum*	1
1928	*Trichophyton violaceum*	1
1929	*Trichostrongylus colubriformis*	1
1922	*Trichuris trichiura*	1
1922	*Trichuris trichiura*	3
2014	*Tropheryma whipplei*	1
2014	*Trypanosoma lewisi*	1
1907	varicella-zoster virus	3
2015	varicella-zoster virus	2
1893	variola virus	3
1874	*Vibrio cholerae*	3
1925	*Vibrio cholerae*	1
1998	*Vibrio parahaemolyticus*	1
1979	*Wuchereria bancrofti*	1
1915	*Yersinia pestis*	3
1917	*Yersinia pestis*	1
2016	Zika virus	1

Table 2. Total number of pathogens observed in Laos between 1974 and 2016 by taxa

Domain	Bacteria	Viruses	Protozoa	Fungi	Arthropods	Helminths
**Totals**	101	58	14	10	3	58

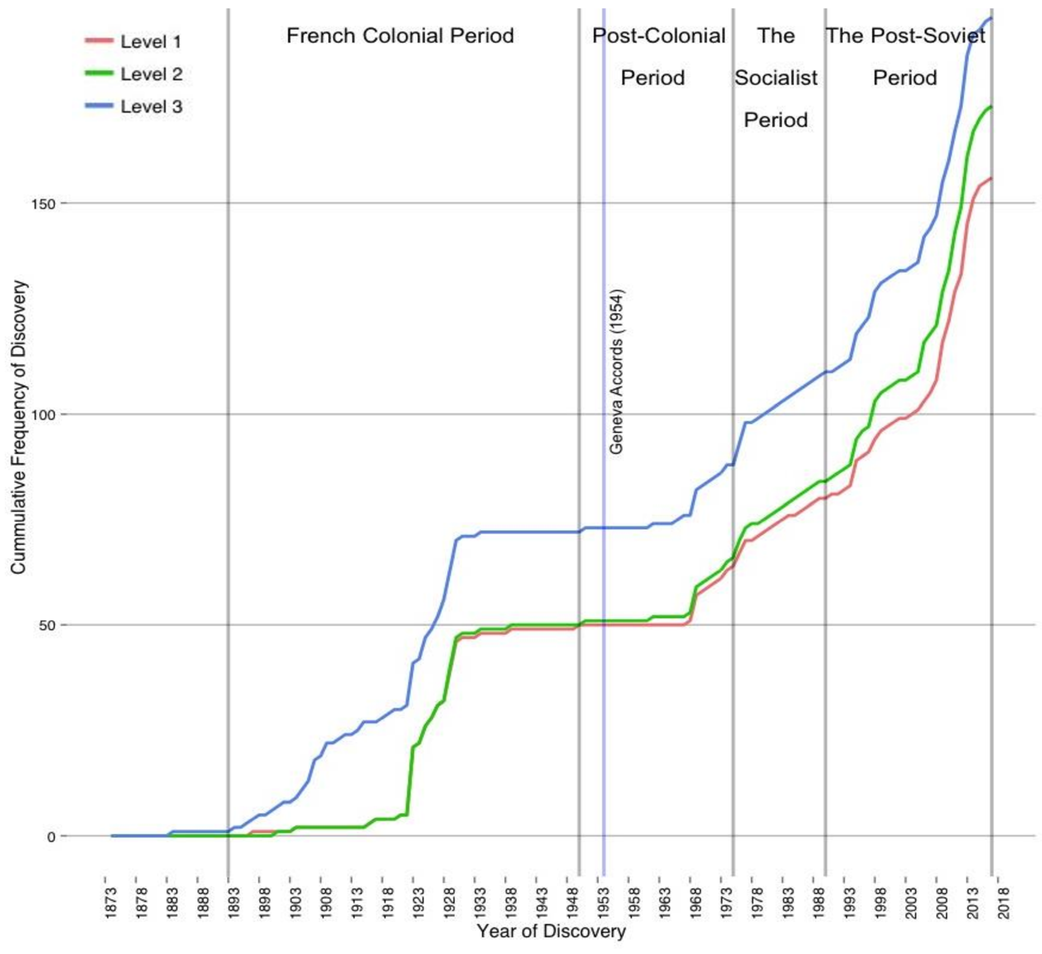

Figure 2: The cummulative frequency of human pathogen discovery in Laos for the period 1874 to 2017 layered by level, where Level 1 represents only culture, molecular (which includes PCR diagnosis or direct microscope observation) discovery, Level 2 is representative of the first discoveries with either a chemical or immune reaction based diagnosis or culture, molecular discovery and, Level 3 is representative of the first discoveries made at any grade of evidence. The vertical blue line identifies the year 1954, the year of the Geneva accords.

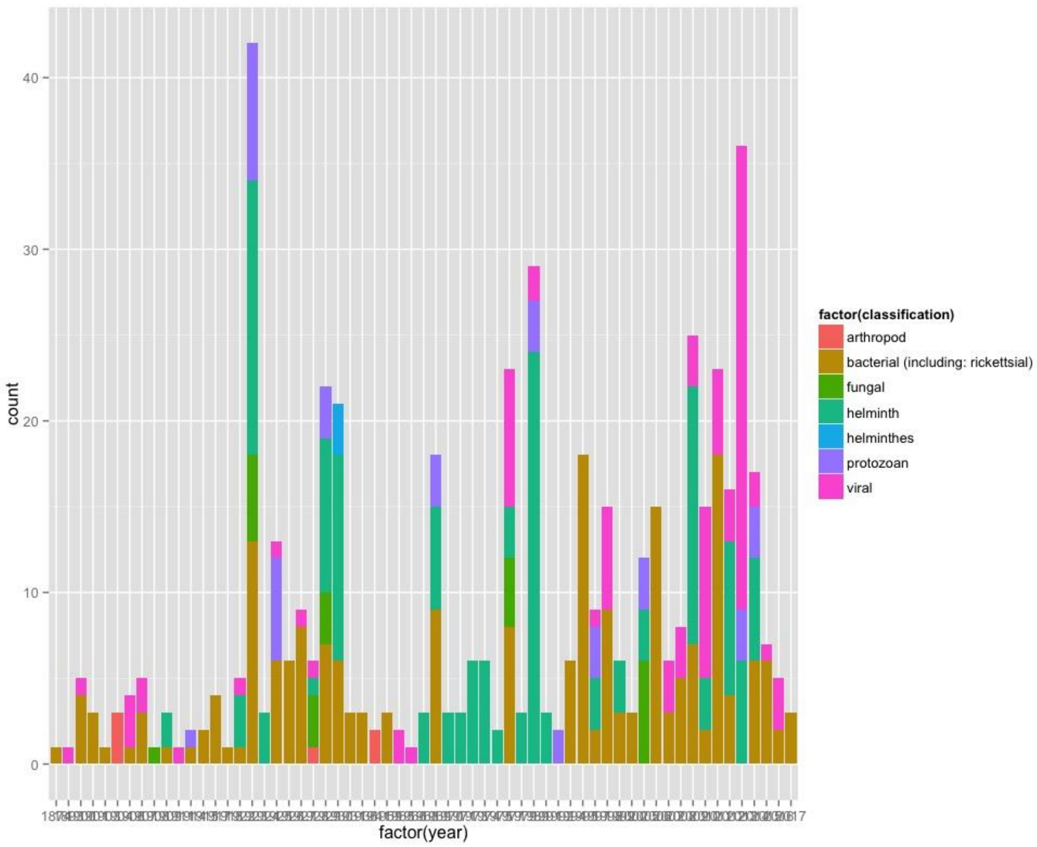

Figure 3 : Frequency of discoveries by year and classification

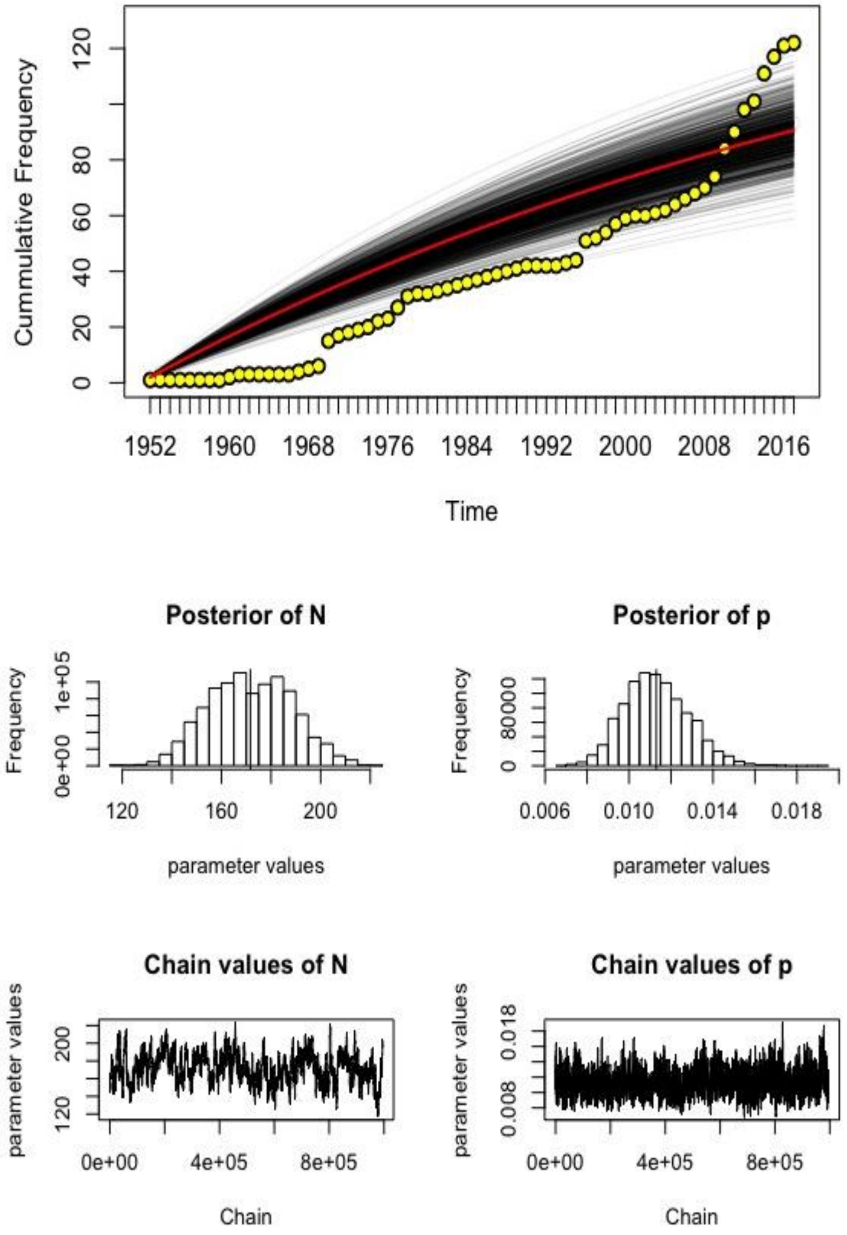

Figure 4: Discovery curve Model for level I data and MCMC output the yellow points represent the observed data, the red line is the best fitting model for a constant discovery rate and the black-lines represent the uncertainty in the model estimates. Underneath the model are a series of histograms which represent the posterior distributions of parameter estimates.

The chain output can be seen at the bottom of the figure and represent the range of parameter values tested and the number of iterations.
